# Using the PRECEDE Model in Understanding Determinants of Quality of Life Among Iranian Male Addicts

**DOI:** 10.5539/gjhs.v6n6p19

**Published:** 2014-06-24

**Authors:** Behzad Karami Matin, Farzad Jalilian, Mehdi Mirzaei Alavijeh, Hossein Ashtarian, Mohammad Mahboubi, Ali Afsar

**Affiliations:** 1 Substance Abuse Prevention Research Center, Kermanshah University of Medical Sciences, Kermanshah, Iran; 2 Social Determinants of Health Research Center, Yasuj University of Medical Sciences, Yasuj, Iran; 3 Health Services Administration, Kermanshah University of Medical Sciences, Kermanshah, Iran; 4 Abadan School of Medical Sciences, Abadan, Iran; 5 Department of Public Health, School of Health, Hamadan University of Medical Sciences, Hamadan, Iran

**Keywords:** Quality of Life, opiate, addict, male, PRECEDE model, Iran

## Abstract

Quality of Life (QOL) in opiate-addicted patients who are receiving methadone maintenance therapy is one of the important issues to be considered in the treatment of addiction. To determine a needs assessment using the PRECEDE model to find out factors related to QOL among Iranian male opiate addicts. This cross-sectional study was conducted in Kermanshah, Iran in 2013. A total of 762 male opiate addicts, who were referred to addiction treatment centers for receiving methadone maintenance treatment, were randomly selected to participate voluntarily in the study. SF-12, predisposing factors, enabling factors, reinforcement factors, and methadone maintenance treatment intention were used to find the related factors. Data were analyzed by the SPSS software (ver. 21.0) using the t-test, one-way analysis of variance (ANOVA), bivariate correlations, and linear regression at 95% significant level. Linear regression analysis showed the determinant variable accounted for 17% of the variation in QOL. Our findings suggest, providing social support for addicts could be beneficial results for the increasing quality of life among them.

## 1. Introduction

Substance addiction is one of the major worldwide problems that destroys economy, familial relationships, and the abuser’s career and has several side effects (Abdollahi et al., 2014). Drug abuse in community is so important that has attracted the attention of different groups such as physicians, psychologists, sociologists, and police forces (Amiri et al., 2013). Addiction is defined as a chronic, compulsive, or uncontrollable drug use to the extent that a person (an addict) cannot or will not stop the use of some drugs. It usually implies a strong psychological dependence as well as physical dependence resulting in withdrawal syndrome when use of the drug is stopped (Jalilian et al., 2014).

The physical environment and social development of urban areas has led to social and cultural heterogeneity. This, in turn, has a considerable influence on social difficulties seen in urban areas. The type of accommodation, their surrounding social milieu, poorly paid jobs, social failures, and economic poverty paves the way for social harms such as drug addiction (Amiri et al., 2013). Furthermore, currently, more than 26 million annual deaths occur due to drug use worldwide ant it will increase to more than 40 million within the next 20 years, of which more than a third will occur in developing countries (Ahmadpanah et al., 2013). In this regard, Iran regarding its specific geographical, social, and cultural situations is assumed a vulnerable environment for drug abuse. Research showed that 1.2 to 2 million persons in Iran are addicts and 11 million persons are struggling with their own addiction or addiction in a member of their family (Jalilian et al., 2014). Former studies have noted that substance abuse is spreading in Iran (Mirzaei Alavijeh et al., 2013a, 2013b; Jalilian et al., 2013a). According to development of new perspective to an addicted person (addiction is called a disease) in the community, establishing and equipping centers which can include specialists for addiction treatment has become necessary (Parvizi et al., 2009). Besides, monitoring Quality of Life (QOL) in addicted patients has gained special attention in clinical research (Jalilian et al., 2012). The concept of QOL is relatively new in behavioral research, especially among addicted persons. Historically, when assessing well-being, the substance use disorder field has used the ‘health-related quality of life’ (HRQOL) measurement model, i.e., a patient’s perception of how his or her health status affects physical, psychological, and social functioning and well-being (Tracy et al., 2012).

These facts necessitate execution of health interventions. On the other hand, to design a curriculum, studies have shown that the most effective curricula are based on theory-based approaches which have originated from behavior change patterns and selection of a suitable pattern of theory of health education is the first step for processing of a curriculum and health education depends on proficiency in use of the best theories and suitable strategies based on each event (Glanz, Rimer, & Viswanath, 2008). The PRECEDE-PROCEED model is a participatory model for designing and implementing health intervention program; this model was presented by Green and Kreuter. PRECEDE stands for predisposing, reinforcing, and enabling constructs in education/environmental diagnosis and evaluation (Green & Kreuter, 2005). In addition, one of the stages in PRECEDE-PROCEED model is educational and organizational diagnosis step. This step is arguably the most difficult in the planning process, yet it is without question a vital step; the overall task here is to develop a plan designed to achieve the program objective (Crosby & Noar, 2011). One of the health program planning models for QOL assessment is PRECEDE-PROCEED model; this model is a health program planning model with participatory research principles that has been used in a variety of communities to optimize health (Walsh et al., 2014).

The main objective of this study was to educational need assessment to understand the development of an intervention to improve QOL in a sample of Iranian male addicts referred to addiction treatment centers in Kermanshah County, the west of Iran based on PRECEDE model.

## 2. Methods

### 2.1 Participants and Procedure

The study was part of a project conducted among male addicts in Kermanshah during 2013, with the goal of providing knowledge in order to raise QOL among addicts. The participants were male addicts who were referred to addiction treatment centers for receiving methadone maintenance treatment. Six hundred and ninety-eight (91%) participants out of 762 subjects signed the consent form and voluntarily agreed to participate in the study. The study protocol was approved by the institutional review board at the Kermanshah University of Medical Sciences, Kermanshah, Iran. The participants’ age ranged from 20 to 50 years with a mean age of 31.40 (SD = 7.25) years. The sample size was calculated at 95% significant level according to the results of a pilot study and a sample of 762 was estimated to be sufficient.

To enroll the participants and collect the required data, the following stages were done. First, different areas of the Kermanshah city were classified based on the division of the geographical region; next for each social class two addiction treatment centers were randomly selected. Then, subjects referred to the addiction treatment centers for taking methadone maintenance treatment, were enrolled into this study voluntarily. Finally, the volunteers were given the self-report questionnaire to be filled out by them. For volunteers who reported literate education, the required information was collected through conducting an interview.

### 2.2 Pilot Study

In order to achieve, the factors affecting the quality of life among participants, a research team carried out a comprehensive literature review as well as interviews with 30 male addicts referred to addiction treatment centers in Kermanshah County. Data analysis showed, factors such as (attitude toward methadone maintenance treatment, perceived behavior control not to use the drug, spending money, lack of time, friend support, family support, social support, methadone maintenance treatment intention) were effective on quality of life. These factors were classified in three categories (predisposing factors, enabling factors, and reinforcement factors). Prior to conducting the main project, a pilot study was conducted to assess the content validity of the questionnaires. The pilot study participants comprised 30 male addicts, similar to those who participated in the main study. The pilot study was conducted in order to obtain feedback about the clarity, length, comprehensiveness, and required completion time of the study questionnaire as well as to collect data to estimate the internal consistency of the questionnaires.

### 2.3 Demographics

Background data collected included age, marital status (single, married, divorce, widower) the age at which the subject started drug abuse, and economic status (independent, dependent).

### 2.4 Measures

#### 2.4.1 Health-Related Quality of Life (SF-12) Scale

This scale which has health-related quality of life 12 items (SF-12) was used here (Selim et al., 2009). SF-12 yields two scores: the physical component summary (PCS) and the mental component summary (MCS). In the calculation of the MCS and PCS scores, each item makes a contribution to each score according to a pre-specified weight. The reliability coefficient for this scale in our study was 0.85, suggesting that the internal consistency was adequate.

#### 2.4.2 Predisposing Factors Scale

The items which assessed components of the predisposing factors were designed based on standard questionnaires applied to methadone maintenance treatment (Allahverdipour et al., 2012; Lai, 2013; Jalilian et al., 2013b). Predisposing factors were measured by 9 items. This section included two parts: attitude toward methadone maintenance treatment and perceived behavior control not to use the drug. Five items measured specifically attitudes toward methadone maintenance treatment (e.g., if I taking methadone maintenance treatment, it would help me to better communicate with people around me). Four other items measured the perceived behavioral control not to use the drug (e.g., I believe that I can manage myself against pressure of using a drug). The reliability coefficient for the predisposing factors scale was 0.92, suggesting that the internal consistency was good.

#### 2.4.3 Enabling Factors Scale

Enabling factors related to methadone maintenance treatment were measured by six items based on a questionnaire designed by the research team, for example: “taking methadone maintenance treatment, need to spending money”. Cronbach’s alpha for the enabling factors scale was 0.83.

#### 2.4.4 Reinforcement Factors Scale

For measuring reinforcement factors, we used social support scale; in addition, social support was evaluated by a 12-item standard scale (Canty-Mitchell & Zimet, 2000). Each item was measured on an ordinal 5-point Likert-type scale (1 = strongly disagree, 5 = strongly agree). Multidimensional scale of perceived social support included three scopes (family, friend, and other significant). Example of the items is: “There is a special person who is around when I am in need”. The reliability coefficient for the social support scale in our study was 0.89, suggesting that the internal consistency was adequate. The reliability coefficient for the reinforcement factors (social support scale) in our study was 0.81, suggesting that the internal consistency was adequate.

#### 2.4.5 Methadone Maintenance Treatment Intention Scale

Behavioral intention to receive methadone maintenance treatment intention was measured by two items based on a standard questionnaire (Allahverdipour et al., 2012; Lai, 2013; Jalilian et al., 2013b). For example: “I intend not to use the drug forever”. Cronbach’s alpha for the behavioral intention scale was 0.75.

### 2.5 Statistical Analysis

Data were analyzed by the Statistical Package for the Social Sciences (SPSS) version 21.0 using bivariate correlations, t-test, and one-way analysis of variance (ANOVA) tests at 95% significant level.

## 3. Results

Mean initiation age for drug use was 15.62 years (range, 10-24 years). Demographic characteristics of the participants are shown in Table 1.

**Table 1:** Distribution of the demographic characteristics among the participants

Variables	Number	Percent
Age group (year)		
20-30	340	48.7
31-40	269	38.5
41-50	89	12.8

Initiation age for drug use (year)		
10-14	266	38.1
15-19	306	43.8
20-24	126	18.1

Education level		
Illiterate	137	19.6
Primary school (5 grades)	141	20.2
Secondary school (8 grades)	234	33.5
High school (12 grades)	115	16.5
Academic	71	10.2

Marital Status		
Married	343	49.1
Single	240	34.4
Divorced	115	16.5

Economic Status		
Independent	211	30.2
Dependent	487	69.8

**Table 2:** Association between background variable and quality of life

	Variable	Mean	SD	P
Education level	Illiterate	59.36	7.42	P <0.001
	Primary school	54.23	8.27	
	Secondary school	60.65	11.95	
	High school	54.08	4.59	
	Academic	61.53	12.44	

Marital Status	Married	59.22	9.09	0.011
	Single	57.32	12.41	
	Divorced	56.43	5.87	

Economic Status	Independent	65.09	12.24	P <0.001
	Dependent	55.08	7.01	

In addition bivariate associations among the age, initiation age for drug use, years engaged in drug abuse and quality of life showed statistically significant at either 0.01 level. The results showed that QOL was associated with the age (r = 0.132), and initiation age for drug use (r = 0.492), while inversely correlated with years engaged in drug abuse (r = -0.121).

Table 3 shows bivariate correlations between the PRECEDE variable and QOL. All of them were statistically significant at 0.01.

**Table 3:** Correlation between different PRECEDE variable and quality of life

	Mean (SD)	Scores Range	X^1^	X^2^	X^3^	X^4^
X^1^. Predisposing Factors	30.24 (6.86)	9-45	1			
X^2^. Enabling Factors	18.09 (4.74)	6-30	0.494	1		
X^3^. Reinforcement Factors	34.66 (8.40)	12-60	0.530	0.675	1	
X^4^. Behavioral Intention	5.68 (1.77)	2-10	0.504	0.684	0.711	1
X^5^. Quality of Life	58.11 (10.03)	0-100	0.201	0.290	0.399	0.366

Linear regression analysis was performed to explain the variation of QOL. As shown in Table 4, collectively, PRECEDE variables accounted for 17% of the variation seen in QOL.

**Table 4:** PRECEDE variables which were predictor of quality of life

Variable	Unstandardized Coefficients		Standardized Coefficients	t	Sig
	B	SE B	Beta		
Step 1					
Predisposing Factors	0.064	0.062	0.044	1.038	0.300
Enabling Factors	0.043	0.109	0.020	0.398	0.691
Reinforcement Factors	0.364	0.064	0.305	5.642	0.001
MMT Intention	1.043	0.304	0.185	3.430	0.001

Step 2					
Predisposing Factors	0.068	0.061	0.046	1.110	0.267
Reinforcement Factors	0.356	0.061	0.298	5.828	0.001
MMT Intention	0.999	0.283	0.177	3.530	0.001

Step 3					
Reinforcement Factors	0.336	0.059	0.282	5.748	0.001
MMT Intention	0.932	0.276	0.165	3.371	0.001

*R^2^=0.17, F=72.671, P <0.001*					

## 4. Discussion

Our result showed that most participants (48.7%) were young people who aged less than 30 years. Mean age at which they started drug abuse was just 15.62 years. Previous studies in this context among Iranian population indicate that during the last two decades onset age of drug abuse has decreased and sometimes has reported as eight years (Shamsi Meymandi, 2008). In this regard, Jalilian et al. Reported initiation age of drug use among male addicted persons who were in prison as 14.36 in a study performed in western part of Iran (Jalilian et al., 2013a).

Addiction is an important worldwide public health problem with various adverse effects (Bakhshani & Hossienbor, 2013). Substance abuse start at an early age could follow greater problems such as conducting crime later in life (Ataee et al., 2014). Considering that the age of drug addiction has decreased significantly, it seems that implementing behavioral interventions for younger children is necessary.

Another finding of our study which warrants attention is the low educational level among respondents. In fact, about 90% had educational level of high school diploma or lower level. This is in line with the findings of earlier studies among Iranian addicts (Jalilian et al., 2014).

The QOL has been acknowledged as an important instrument in the evaluation of drug program (Giacomuzzi, et al., 2003). The study findings also indicated the, positive correlation between age, onset age drug use and quality of life. In addition, inversely correlated with years engaged in drug abuse and quality of life; these could be deduced that with increasing years of involved in drug addiction and complications develop. Our result also indicated higher QOL among patients with higher level of education. In this regard, several study reported that QOL have a positive correlation with education (Meiavia et al., 2013; Anu et al., 2014; Kirchhoff et al., 2014; Mazloomy Mahmoudabad, 2011). Furthermore, our findings indicated high level of QOL among married participants; this result may be because of better social support status in married people.

Bivariate associations between QOL predictors indicated that the reinforcement factor (in our study social support), methadone maintenance treatment intention, enabling factor to maintenance treatment and reinforcement factor (in our study perceived behavior control not to drug use and attitude toward maintenance therapy) had a statistically significant direct correlation. Furthermore, a hierarchical multiple regression analysis showed social support and methadone maintenance treatment intention were stronger predictor factors for QOL among drug addicts, respectively. In this regard, Lakey and Cohen presented three theoretical perspectives on the relationship between social support and health: the stress and coping perspective, the social constructionist perspective, and the relationship perspective (Lakey & Cohen, 2000). Perceived social support, with enhancement of individual’s mental health, services like a shield against drug relapse (Dodge & Potocky, 2000). Much research in substance abuse field reported positive roles for social support in drug cessation, maintenance therapy, and also increasing QOL (Davis & Jason, 2005; Warren et al., 2007; Copello, 2000; Rao et al., 2012; Ponizovsky & Grinshpoon, 2007; Maremmani, 2007). For example, Jalilian et al. carried out a research on male addicts in Kermanshah, Iran during 2010 and reported that family support was a more important effect among types of social supports in drug cessation. They recommended providing educational programs to addicts’ families for more support of patients at drug cessation (Jalilian et al., 2014). In addition, Rao et al. in their study among people living with HIV in Beijing, China reported perceived social support was associated with fewer stigmas, fewer depressive symptomatology, and better QOL (Rao et al., 2012). Furthermore, Ponizovsky and Maremmani, in their studies reported beneficial effects of maintenance treatment programs to satisfaction with QOL (Ponizovsky & Grinshpoon, 2007; Maremmani, 2007).

Since social support and behavioral intention to maintenance therapy in promoting QOL among addicts have been shown, reinforced social support to addicts, and determined factors related with maintenance therapy should be considered more.

Attention to QOL of addicted patients during receiving methadone maintenance therapy is important to understand how different dimensions of QOL are improved during this period. This also enables us to find out how addicts contribute to be abstinent and decrease the dosage of drug abused (Feelemyer et al., 2014). There are many factors that effect on QOL of drug abusers. I Identification of these factors can be helpful for designing a training program in order to improve the QOL of such patients and consequently, improve their health. Our findings suggest that providing social support for addicts could be beneficial to raise their QOL.

## 5. Limitations

Though our research has numerous strengths such as theory driven and high sample size, the findings reported in this study had certain limitations. First, the use of two methods for data collection (self-reporting and interview) raises concerns in terms of recall bias. Second, data collection was done only among addict persons who were referred to addiction treatment centers for receiving methadone maintenance treatment and other groups of drug users were not included. This may limit the generalizability of the findings. To overcome this limitation, we suggest that in future studies comprehensive information be collected from drug users in other treatment groups as well such as narcotics anonymous (NA) and therapeutic community (TC). This method of data gathering will also enable the researchers to compare QOL among a more diverse population of addicted patients.

## References

[ref1] Abdollahi Z, Taghizadeh F, Hamzehgardeshi Z, Bahramzad O (2014). Relationship between Addiction Relapse and Self-Efficacy Rates in Injection Drug Users Referred to Maintenance Therapy Center of Sari, 1391. Global Journal of Health Science.

[ref2] Ahmadpanah M, Mirzaei Alavijeh M, Allahverdipour H, Jalilian F, Afsar A, Gharibnavaz H (2013). Effectiveness of coping skills education program to reduce craving beliefs among addicts referred to addiction centers in Hamadan: a randomized controlled trial. Iranian Journal of Public Health.

[ref3] Allahverdipour H, Jalilian F, Shaghaghi A (2012). Vulnerability and the intention to anabolic steroids use among Iranian gym users: An application of the theory of planned behavior. Substance use & misuse.

[ref4] Amiri M, Dejman M, Dastoury M, Roushanfekr P (2013). The Relationship between Addiction and Socio-demographic Characteristics of Iranian Newcomer Prisoners. Global Journal of Health Science.

[ref5] Anu V. K, Pushpa P, Kumar S. S (2014). Quality of Life of Patients undergong Haemodialysis at BP Koirala Institute of Health Sciences. Journal of Manmohan Memorial Institute of Health Sciences.

[ref6] Ataee M, Mousaviraja A, Ahmadi Jouybari T, Jalilian F, Amoei M. R, Allahverdipour H, Mahboubi M (2014). Assessing reciprocal relation between drug abuse and crime: a cross-sectional study among prisoners kermanshah county, the west of Iran. Journal of Science and Today’s World.

[ref7] Bakhshani N. M, Hossienbor M (2013). A comparative study of self-regulation in substance dependent and non-dependent individuals. Global Journal of Health Science.

[ref8] Canty-Mitchell J, Zimet GD (2000). Psychometric properties of multidimensional scale of perceived social support in urban adolescents. American Journal of Community Psychology.

[ref9] Copello K (2000). Social Role Negotiation Skills For substance abusing adolescents: A Group model. Journal of Substance Abuse Treatment.

[ref10] Crosby R, Noar S. M (2011). What is a planning model? An introduction to PRECEDE-PROCEED. Journal of public health dentistry.

[ref11] Davis M. I, Jason L. A (2005). Sex differences in social support and self-efficacy within a recovery community. American Journal of Community Psychology.

[ref12] Dodge K, Potocky M (2000). Female substance abuse: characteristics and correlates in a sample of inpatient clients. Journal of Substance Abuse Treatment.

[ref13] Feelemyer J. P, Jarlais D. C. D, Arasteh K, Phillips B. W, Hagan H (2014). Changes in quality of life (WHOQOL-BREF) and addiction severity index (ASI) among participants in opioid substitution treatment (OST) in low and middle income countries: An international systematic review. Drug and alcohol dependence.

[ref14] Giacomuzzi S. M, Riemer Y, Ertl M, Kemmler G, Rössler H, Hinterhuber H, Kurz M (2003). Buprenorphine versus methadone maintenance treatment in an ambulant setting: a health-related quality of life assessment. Addiction.

[ref15] Glanz K, Rimer B. K, Viswanath K (2008). Health behavior and health education: Theory, research, and practice.

[ref16] Green L. W, Kreuter M. W (2005). Health program planning: an educational and ecological approach.

[ref17] Jalilian F, Zinat Motlagh F, Amoei M. R, Hatamzadeh N, Gharibnavaz H, Mirzaei Alavijeh M (2014). Which One Support (Family, Friend or Other Significant) is Much More Important to Drug Cessation? AStudy among Men Kermanshah Addicts, the West of Iran. J Addict Res Ther.

[ref18] Jalilian F, Mirzaei Alavijeh M, Amoei M. R, Zinat Motlagh F, Hatamzadeh N, Allahverdipour H (2013a). Prevalence and pattern of drug abuse among prisoners in Kermanshah city. Journal of Health Education and Health Promotion.

[ref19] Jalilian F, Mirzaei Alavijeh M, Emdadi S. H, Nasirzadeh M, Barati B, Hatamzadeh N (2012). The quality of life of women with type 2 diabetes: the study of self-efficacy. Health System Research.

[ref20] Jalilian F, Karami-Matin B, Mirzaei Alavijeh M, Ataee M, Mahboubi M, Motlagh F, Aghaei A (2013). Prevalence and Factor Related to Ritalin Abuse among Iranian Medical College Student: An Application of Theory of Planned Behavior. TERAPEVTICHESKII ARKHIV.

[ref21] Kirchhoff A. C, McFadden M, Warner E. L, Kinney A. Y (2014). Quality of Life and Comorbidities Impact Education and Employment for Survivors of Adolescent and Young Adult Cancers. Cancer Epidemiology Biomarkers & Prevention.

[ref22] Lai Y. H (2013). Factors associated with methadone maintenance treatment among injecting drug users. International Journal of Health Research and Innovation.

[ref23] Lakey B, Cohen S, Cohen S, Underwood L. G, Gottlieb B. H (2000). Social support a theory and measurement. Social support measurement and intervention: A guide for health and social scientists.

[ref24] Maremmani I, Pani P. P, Pacini M, Perugi G (2007). Substance use and quality of life over 12 months among buprenorphine maintenance-treated and methadone maintenance-treated heroin-addicted patients. Journal of substance abuse treatment.

[ref25] Mazloomy Mahmoudabad SS, Zolghadr R, Mirzaie Alavijeh M, Hasan Baigi A (2011). Relationship between Chronic Stress and Quality of Life in Female Students in Yazd City in 2011. Toolo-e-Behdasht.

[ref26] Meiavia A, Marques D, Gomes A. A (2013). Quality of sleep and quality of life in higher education students. Sleep Medicine.

[ref27] Mirzaei Alavijeh M, Jalilian F, Mazloomy S, Movahed E, Motlagh F, Hatamzadeh N (2013a). Predictors of drug abuse among students with application of Prototype/Willingness Model. Journal of Police Medicine.

[ref28] Mirzaei Alavijeh M, Mazloomy S, Yassini S, Askarshahi M, Jalilian F, Zinat Motlagh F (2013b). Fathers’ behavioral intention and behavior in prevention of children tendency toward addictive drugs. Journal of Health Education and Health Promotion.

[ref29] Parvizi D, Rahgozar M, Vamegi R, Foroghan M (2009). Influencing factors on client satisfaction in governmental addiction treatment centers and comparison with private centers in Kordestan province. HAKIM.

[ref30] Ponizovsky A. M, Grinshpoon A (2007). Quality of life among heroin users on buprenorphine versus methadone maintenance. The American Journal of drug and alcohol abuse.

[ref31] Rao D, Chen W. T, Pearson C. R, Simoni J. M, Fredriksen-Goldsen K, Nelson K, Zhang F (2012). Social support mediates the relationship between HIV stigma and depression/quality of life among people living with HIV in Beijing, China. International journal of STD & AIDS.

[ref32] Selim A. J, Rogers W, Fleishman J. A, Qian S. X, Fincke B. G, Rothendler J. A, Kazis L. E (2009). Updated US population standard for the Veterans RAND 12-item Health Survey (VR-12). Quality of Life Research.

[ref33] Shamsi Meymandi M, Ziaeddini H, Sharifi Yazdi A (2008). Opinion of high school students of Kerman towards affecting factors on narcotics tendency. JQUMS.

[ref34] Tracy E. M, Laudet A. B, Min M. O, Kim H, Brown S, Jun M. K, Singer L (2012). Prospective patterns and correlates of quality of life among women in substance abuse treatment. Drug and alcohol dependence.

[ref35] Walsh J. R, White A. A, Kattelmann K. K (2014). Using PRECEDE to Develop a Weight Management Program for Disadvantaged Young Adults. Journal of nutrition education and behavior.

[ref36] Warren J. I, Stein J. A, Grella C. E (2007). Role of social support and self-efficacy in treatment outcomes among clients with co-occurring disorders. Drug and Alcohol Dependence.

